# Histamine metabolism influences blood vessel branching in zebrafish *reg6 *mutants

**DOI:** 10.1186/1471-213X-8-31

**Published:** 2008-03-25

**Authors:** Cheng-chen Huang, Chin-Wei Huang, Yih-Shyun E Cheng, John Yu

**Affiliations:** 1 Institute of Cellular and Organismic Biology, Academia Sinica, Taipei, Taiwan; 2 Genome Research Center, Academia Sinica, Taipei, Taiwan

## Abstract

**Background:**

Vascular branching morphogenesis is responsible for the extension of blood vessels into growing tissues, a process crucial for organogenesis. However, the genetic mechanism for vessel branching is largely unknown. Zebrafish *reg6 *is a temperature-sensitive mutation exhibiting defects in blood vessel branching which results in the formation of swollen vessel lumina during capillary plexus formation.

**Results:**

We performed a screening for chemical suppressors of *reg6 *and identified SKF91488, an inhibitor of histamine methyltransferase (HMT), that can rescue the *reg6 *vessel branching defects in a dose-dependent manner. Inhibition of HMT by SKF91488 presumably blocks histamine degradation, thus causing histamine accumulation. Consistent with this idea, we found that a high level of histamine also showed significant suppression of *reg6 *vessel phenotypes. Interestingly, when *reg6 *adults that had already developed swollen vessel lumina in regenerating fins were treated with histamine or SKF91488, either treatment significantly reduced the number of swollen vessels within 12 h, suggesting a rapid and constant influence of histamine on blood vessel branching. Furthermore, the expression of HMT was significantly elevated in *reg6 *regenerating fins. Conversely, lowering histamine by administering urocanic acid, a histidine decarboxylase inhibitor, enhanced the *reg6 *phenotypes. Finally, we identified that the transcription factor, egr-1 (early growth response factor 1), was closely associated with the *reg6 *phenotype and chemical treatments.

**Conclusion:**

Taken together, our results suggest that blood vessel branching is influenced by histamine metabolism, possibly through regulating the expression of the egr-1 transcription factor.

## Background

Blood vessels are important for transporting oxygen and nutrients to cells for survival and proper functioning. In early embryos, the pioneer trunk vessels, the dorsal aorta and cardinal vein, develop from vascular endothelial cells derived from the lateral plate mesoderm through a process called vasculogenesis. Subsequently, blood vessels grow from existing vessels, through a process referred to as angiogenesis, into peripheral tissues and developing organs [1]. In developing embryos, increasing evidence has revealed that blood vessels also provide signals for the proper morphogenesis of developing organs [2]. In the adult form of organisms, angiogenesis and/or vasculogenesis to regrow blood vessels in wounded tissues is needed to repair damaged and replace lost tissues [3,4]. In humans, it has been well documented that angiogenesis plays a vital role in tumor growth, and thus the potential of antiangiogenesis drugs to inhibit tumor growth has been extensively explored worldwide [5].

During angiogenesis, vascular branching is one of the key morphogenetic events, which presumably is responsible for constructing a complex vascular network which assures that blood is available to every single cell. Despite its important role in sustaining organismic development throughout life, the genetic mechanism of blood vessel branching is largely unknown partly due to the intricate interactions between molecular signals and numerous cell types that are involved in angiogenesis, e.g., multiple cellular activities, including cell proliferation, migration, extension, and possibly cell fate specification all of which require signaling through multiple growth factors such as VEGF, FGF, PDGF, and EGF. Interactions between blood vessel endothelial cells and neighboring cells and even cells in the bloodstream can also regulate angiogenesis [6]. More recently, it has been implied that immune-mediated cells and molecules can influence blood vessel growth in adults. For example, mast cells have been found to aggregate at sites of active angiogenesis under both physiological and pathological conditions [7]. Histamine has been determined to be an angiogenic factor [8,9] and is thought to be directly involved in tumor growth based on findings of high expression levels and enzymatic activity of the key histamine synthesis enzyme, histidine decarboxylase, in numerous growing tumors, such as small-cell lung carcinoma [10,11]. In fact, clinical trials have shown that histamine H2 receptor antagonists, such as cimetidine and ranitidine, increase the survival of gastric and colon cancer patients [12,13]. It has also been reported that histamine regulates angiogenesis at the level of cell proliferation and cell permeability [14,15]. Thus, histamine seems to be involved in multiple cellular and molecular interactions during angiogenesis. It is still unclear whether histamine can influence blood vessel branching during angiogenesis, and if it does, through which intracellular signaling pathway it works.

In this report, we took a chemical genetic approach to reveal the role of histamine in vascular branching using a zebrafish *reg6 *mutation which causes specific vascular branching defects without affecting endothelial cell proliferation [16]. We show that vessel branching defects in *reg6 *mutants can be rescued by a high level of histamine either directly added to the water or by inhibiting histamine degradation using a histamine methyltransferase (HMT) inhibitor. On the other hand, blocking histamine synthesis by a histidine decarboxylase inhibitor enhanced the *reg6 *vessel phenotypes. Finally, we show that the expression level of the egr-1 transcription factor, but not the VEGF family, is strongly correlated with the *reg6 *phenotypes, suggesting that histamine might modulate blood vessel branching by regulating egr-1 levels.

## Results

### Specific blood vessel branching defect of the zebrafish *reg6 *mutation

**Figure 1:**
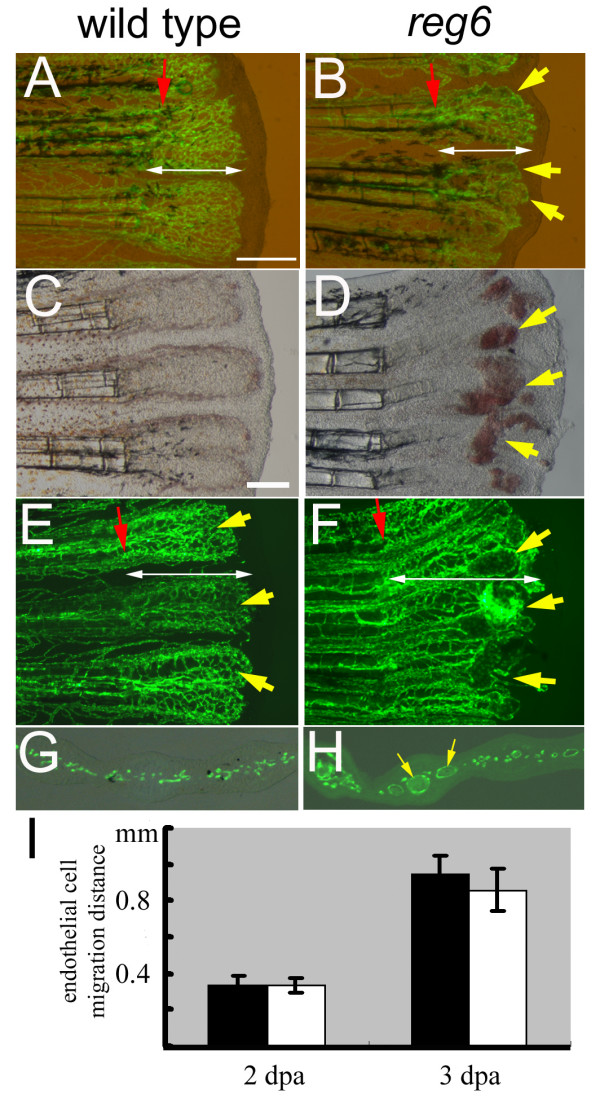
**Normal migration of regenerating endothelial cells in zebrafish *reg6 *mutants**. (A, B) Combined bright field and green fluorescent images of wild-type *TG(fli1:EGFP)*^*y*1 ^(A) and *reg6;TG(fli1:EGFP)*^*y*1 ^(B) regenerating fins at 2 days post amputation (dpa) showing the distance (designated by white double arrows) that the blood vessel endothelial cells (ECs) have migrated. Yellow arrows, dilated vessels of *reg6 *mutants. (C-F) In 3-dpa regenerates, wild-type fish have formed normal capillary plexuses (C, yellow arrows in E)., but *reg6 *fish (D, bright field; F, green fluorescent) have developed numerous swollen vessels (yellow arrows in D and F) resulting in blood cell accumulation (red spots in D). (G, H) Cross-sections of 3-dpa regenerates showing normal regenerating vessels in wild-type (G) and dilated vessels in *reg6 *fish (H, yellow arrows). Red arrows, amputation plane. Scale bars, 300 μm. (I) Quantitative data of the migration distance of regenerating ECs in wild-type (black bars) and *reg6 *fish (white bars) at 2 and 3 dpa.

The zebrafish *reg6 *mutation was isolated by its defects during caudal fin regeneration [17]. It was previously shown that the *reg6 *mutation causes blood vessel dilation during plexus formation of vessel regeneration due to defects in blood vessel branching, not in cell proliferation [16]. Herein, we further tested whether the *reg6 *vessel branching phenotype might be due to or might cause the cell migration defect by measuring the distance endothelial cells were able to migrate at different stages of blood vessel regeneration. Upon amputation, blood vessels undergo wound healing to seal the ends within the first 24 h followed by sprouting to reconnect the severed vessels and resume blood circulation at the amputation site. Blood vessel endothelial cells then proliferate and begin to migrate into the regenerating tissues to form a vascular plexus [16]. We found that regenerating endothelial cells in both wild-type and *reg6 *fish are able to migrate more than 0.35 mm within 2 days post amputation (dpa) (Figure 1A, B, I) when the swollen vessel lumen phenotype has just begun to develop (Figure 1B, yellow arrows), suggesting that vessel dilation is not due to a cell migration defect. When the same fish were measured again at 3 dpa, we found that endothelial cells in *reg6 *fish had migrated more than 0.8 mm, which was similar to that in wild-type fish (Figure 1I) despite significantly swollen vessels having developed (Figure 1D, F, yellow arrows). Thus, the formation of swollen vessel lumina in *reg6 *fish does not seem to cause cell migration defects, at least up to 3 dpa. Cross-sections of *reg6 *regenerating fins revealed enlarged lumina in most of the regenerating vessels (Figure 1H, arrows).

**Figure 2:**
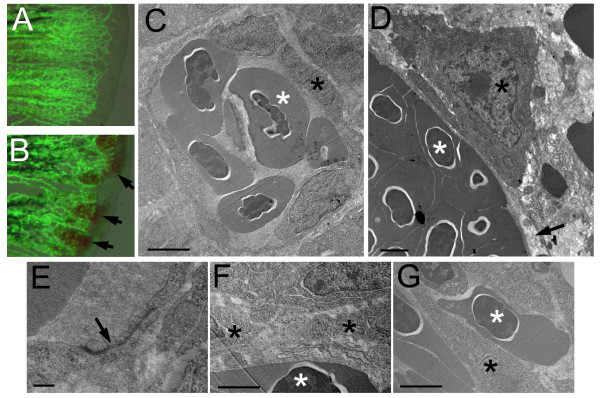
**Cellular defects of *reg6 *endothelial cells**. (A, B) Overlaid images of bright field and green fluorescent protein (GFP) of wild-type (A) and *reg6 *(B) 3-dpa regenerating fins showing the leakage of blood cells in *reg6 *fish (arrows). Transmission electron microscopic images of wild-type fish (C, low magnification; E, high magnification) showing that the blood vessel is enclosed by several endothelial cells which form nice, intact tight junctions (arrow in E). Additional images can be found in Additional file 1. In contrast, the blood vessels in *reg6 *fish (D) were usually leaking as evidenced by the lack of coverage by endothelial cells (arrow) and the presence of blood cells outside blood vessels (as an endothelial cell is sandwiched by two blood cells in G and arrows in Additional file 2). In addition, *reg6 *endothelial cells appeared less stretched-out (D), and tight junction were rarely formed among them (F). Scale bars, 2 μm for C, D, F, and G; 10 nm for E. white asterisks, blood cell; black asterisks, endothelial cell.

To understand the cellular defects in angiogenic endothelial cells of *reg6 *mutants, we examined the cell integrity by whole-mount and transmission electron microscopy (TEM). We first noted that the permeability of *reg6 *endothelial cells seemed defective as leaked blood cells were often observed in *reg6 *regenerating fins (Figure 2B, G). In addition, while endothelial cells stretch thin and form tight junctions and intact enclosed blood vessels of different sizes in wild-type regenerating fins (Figure. 2C, E and Additional file 1), *reg6 *endothelial cells did not appear as stretched-out as those in the wild-type and seldom formed obvious tight junction (Figure 2D, F and Additional file 2). Taken together, our current and previous [16] results suggest that *reg6 *function is specifically required for cell elongation and the formation of tight junctions between endothelial cells that are essential for blood vessel branching morphogenesis.

### Isolation of a chemical suppressor of the *reg6 *mutation

**Figure 3:**
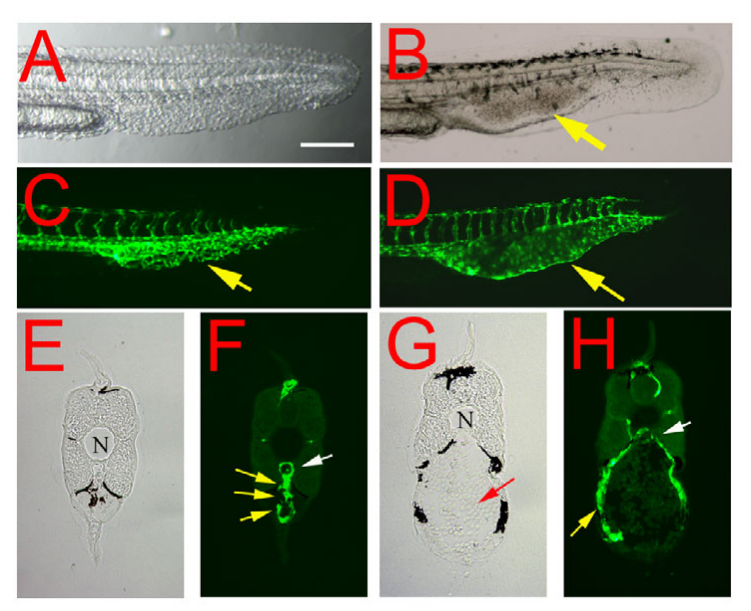
**Embryonic phenotype of the *reg6 *mutation**. (A, C) In a 36-hpf (hours post fertilization) wild-type embryo (A, bright field; C, green fluorescent), the developing caudal veins form vascular plexuses (yellow arrow in C). (B, D) In contrast, endothelial cells of *reg6 *embryos (B, bright field; D, green fluorescent) develop fewer vessel branches and form swollen lumina (yellow arrows). Lateral view; anterior, left; dorsal, top. Scale bar, 100 μm for A-D. (E-H) Cross-sections of the tail regions showing multiple lumina of the capillary plexus of a wild-type caudal vein (E, yellow arrows in F) but a single enlarged lumen in *reg6 *(G, yellow arrow in H). N, notochord; white arrows, dorsal aorta; yellow arrows, caudal vein; red arrow, accumulated blood cells.

**Figure 4:**
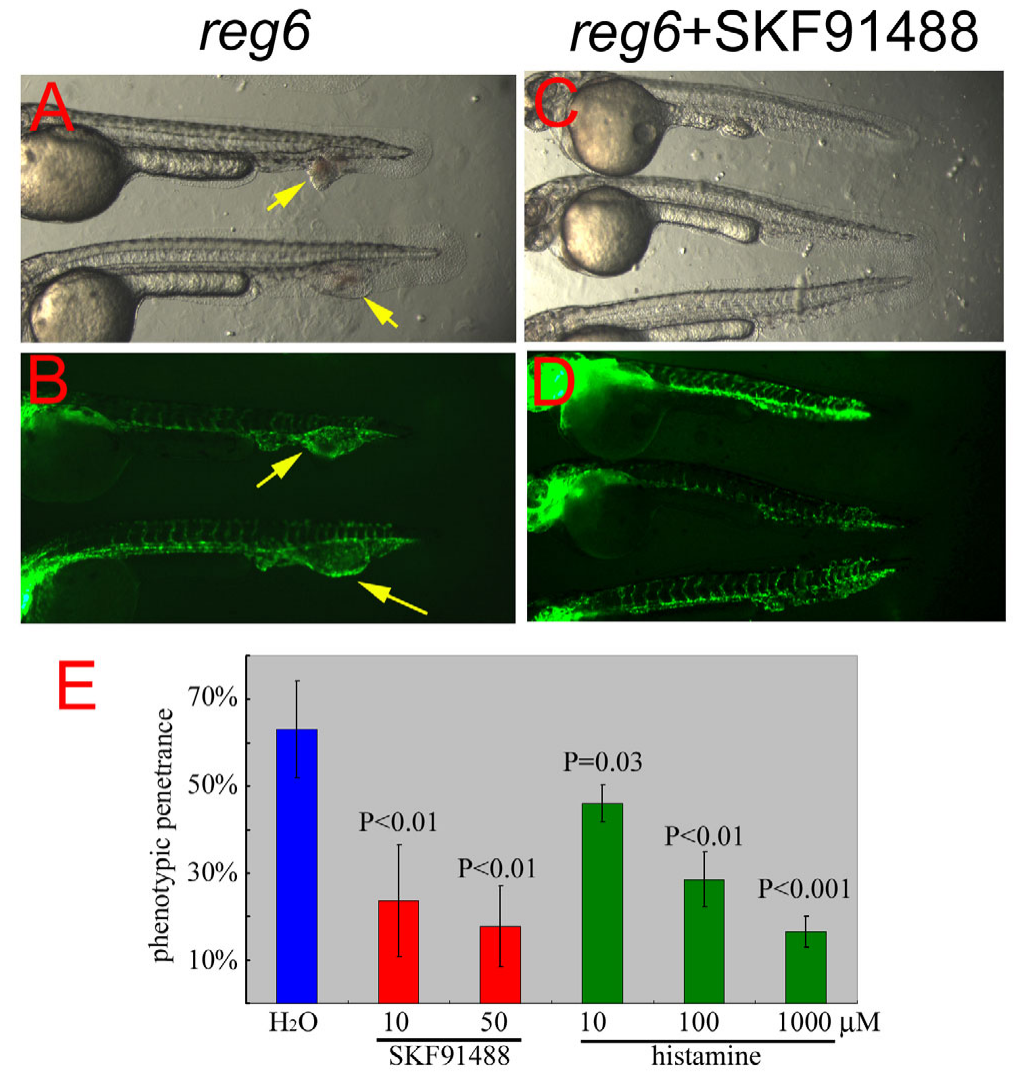
**Suppression of the *reg6 *embryonic phenotype by SKF91488 and histamine**. (A, bright field; B, GFP) Untreated *reg6 *embryos developed dilated caudal veins at 33°C (yellow arrows). (C, bright field; D, GFP) *reg6 *embryos treated with 10 μM HMT inhibitor SKF91488 developed normal caudal veins. Anterior, left; dorsal, top. (E) Typically, almost 70% of *reg6 *embryos formed dilated caudal veins at 33°C (blue bar). The penetrance was reduced by SKF91488 (red bars, *n *= 50) and histamine (green bars, *n *= 50).

A similar swollen-vessel phenotype was also observed in developing caudal veins of *reg6 *mutants during embryogenesis (Figure 3; [16]). In wild-type embryos, the posterior cardinal vein branches to form a transient capillary plexus beginning at around 30 h post fertilization (hpf) (Figure 3A, C; [18]), which is typically located ventral to the dorsal aorta in the tail and is comprised of 2 or 3 parallel yet interconnected vessels (Figure 3E, F). In *reg6 *mutants, however, the caudal vein forms fewer branches and develops a large swollen vascular lumen (Figure 3B, D, G, H). The *reg6 *mutation is temperature sensitive causing up to 70% penetrance of its embryonic phenotypes at 33°C (Figure 4E) and 0%~5% at 20°C (not shown). The temperature sensitivity of its regenerating vessel phenotype is described below (see also [16]). Taking advantage of the incomplete penetrance of the *reg6 *embryonic phenotype, we initiated a screening to search for chemical suppressors and enhancers of *reg6 *mutation. We tested a small collection of known compounds using *reg6*-homozygous embryos (see "Methods"). Of the 560 tested chemicals, one showed significant suppression of *reg6 *phenotypes in developing caudal veins at a concentration of 10 μM (Figure 4C, D). This chemical, 4-(N, N-dimethylamino) butylisothiourea dihydrochloride (SKF91488), is an inhibitor of HMT, the major enzyme responsible for histamine degradation [19]. To test the specificity of SKF91488 on *reg6 *vessel phenotypes and its cytotoxicity to developing embryos, we treated *reg6 *and wild-type embryos with different concentrations of the compound. The results showed that suppression was dose-dependent, and the penetrance of the *reg6 *embryonic phenotype could be reduced to 20% by 50 μM SKF91488 (Figure 4E). The HMT inhibitor caused no morphological defects or vessel over-branching in wild-type embryos at as high as 100 μM (data not shown).

### Suppression of the *reg6 *embryonic phenotype by a high level of histamine

As HMT is responsible for methylating histamine thus precipitating its degradation, the above results led us to hypothesize that SKF91488 might cause accumulation of histamine, which in turn would rescue *reg6 *phenotypes. We tested this hypothesis by treating *reg6 *embryos with histamine. The results showed that a high level of histamine indeed was able to rescue the embryonic phenotypes of *reg6 *mutants, although the concentration of histamine needed to produce significant suppression was 1000 μM (Figure 4E, green bars). Like the HMT inhibitor, a high level of histamine caused no discernible developmental defects in wild-type zebrafish embryos (data not shown).

### Suppression of the *reg6 *regenerating vessel phenotype by SKF91488 and histamine

**Figure 5:**
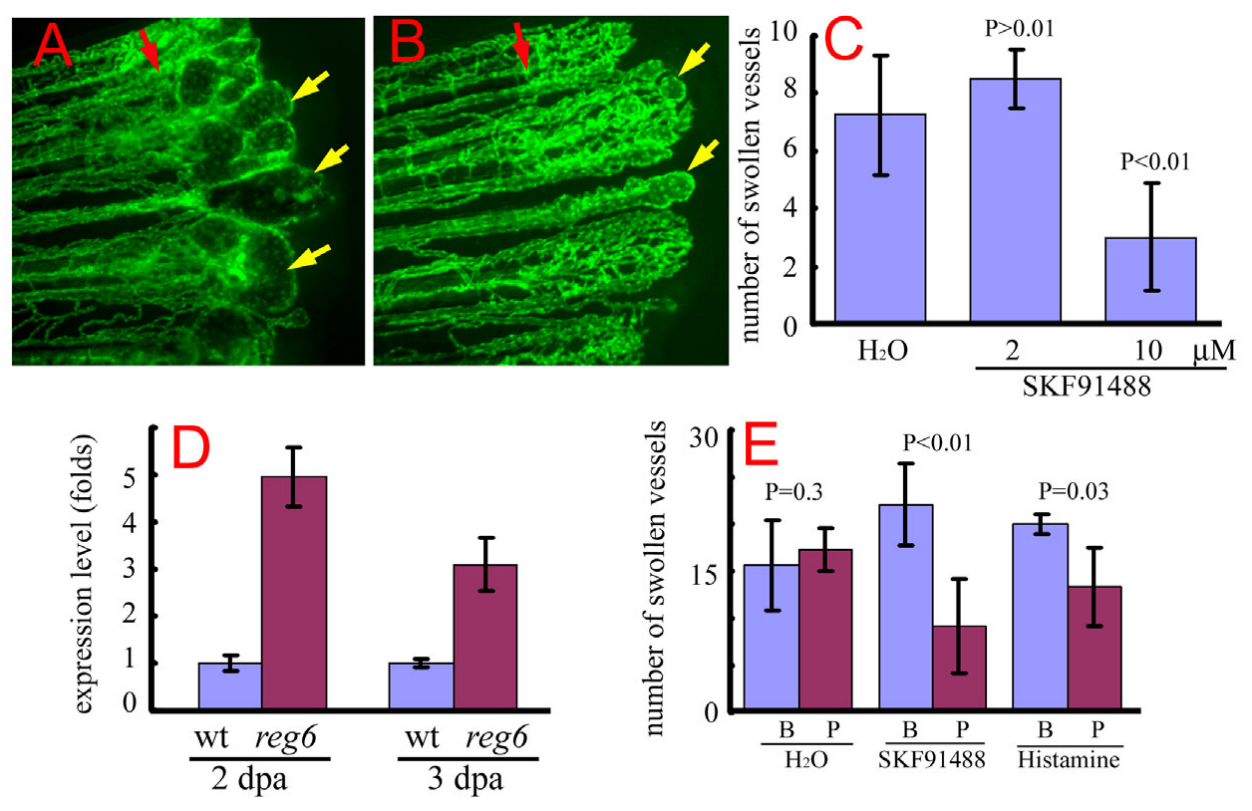
**Suppression of the *reg6 *regenerating vessel phenotype by SKF91488 and histamine**. (A) *reg6 *regenerating vessels formed swollen lumina 3 days post amputation (dpa) at 33°C (yellow arrows). (B) In the presence of 10 μM SKF91488, *reg6 *mutants developed significantly smaller and fewer swollen vessels (yellow arrows). Red arrows, amputation plane. (C) Quantitative data of the suppression of *reg6 *regenerating vessels by SKF91488 (*n *= 5 for each group). This experiment was repeated at least 3 times, and consistent results were observed. (D) Quantitative polymerase chain reaction (QPCR) analyses showed elevated levels of histamine methyltransferase (HMT) RNA in both *reg6 *2 and 3-dpa regenerates compared with the wild-type. (E) Both SKF91488 and histamine could reverse the *reg6 *vessel phenotypes. The number of swollen vessels in *reg6 *mutants was counted at 3 dpa at 33°C (blue bars; B, before treatment) before they were transferred to either fresh water (H_2_O), 10 μM SKF91488, or 1 mM histamine. The number of swollen vessels was counted again 12 h later (red bars; P, post-treatment). Shown is a representative set of data with 10 fish. Similar results were obtained from 2 independent experiments with different stocks of *reg6 *fish.

As shown before, the blood vessel phenotype in regenerating *reg6 *fins develops swollen vessel lumina at a high temperature (33°C). To further confirm the effect of histamine on *reg6 *vessel branching defects, we treated regenerating vessels of *reg6 *and wild-type adult fish with histamine and SKF91488. Consistent with the results obtained from embryos, the regenerated swollen vessel lumen phenotype in *reg6 *fish was both quantitatively and qualitatively suppressed by 10 μM SKF91488, as treated ones formed fewer and smaller blood blisters than their untreated siblings (Figure 5A–C). We then tested whether histamine can also rescue the *reg6 *phenotype in regenerating vessels. In fact, 1 mM histamine did suppress the *reg6 *regenerating vessel phenotype (data not shown). We noted that while most stocks of *reg6 *formed swollen lumina in 15 vessels/fish on average at 33°C (Figure 5E, H_2_O group), some stocks possibly formed 7 swollen vessels/fish (Figure 5C, H_2_O group). But within the same inbred family, the penetrance was less variable (CC Huang, unpublished observations). Therefore, we were careful to use fish of the same stock for making comparisons. These results suggested that increasing the histamine level can rescue the blood vessel branching defect of zebrafish *reg6 *mutants.

The above results led us to hypothesize that the expression of HMT might increase in *reg6 *mutants, which is responsible for the histamine deficit. To test this, we analyzed the amount of HMT RNA in wild-type and *reg6 *regenerates by quantitative PCR (QPCR). We found that the amounts of HMT RNA were nearly 5- and 3-fold higher in *reg6 *2 and 3-dpa regenerates, respectively, than in wild-type ones (Figure 5D).

### Reversion of *reg6 *dilated vessels by histamine and SKF91488

We wondered whether histamine and SKF91488 are able to reduce the severity of the vessel phenotype in *reg6 *mutants even after significant swollen vessels had formed. To test this, we first allowed *reg6 *mutants to regenerate at 33°C for 3 days to develop swollen lumina, and then shifted them to either 10 μM SKF91488, 1 mM histamine, or H_2_O. We then compared the number of swollen vessels before and then 12 h after the shift. In the H_2_O group, the number of swollen vessels in *reg6 *fish was about the same within the 12-h period: near 20 swollen vessels/fish (Figure 5E). With SKF91488 treatment, however, the number of swollen vessels was significantly reduced to an average of 10 swollen vessels/fish (Figure 5E, *P *< 0.01, *n *= 10). Histamine also reduced the number from 20 to fewer than 15 (*P *= 0.03, *n *= 10). In the distal portions of regenerating fins of *reg6 *adults, the vessel defects usually caused necrosis and degeneration, which were thus greatly reduced by histamine and SKF91488 (data not shown). We concluded that a high level of histamine can reverse the swollen vessel phenotype of the *reg6 *mutation.

### Enhancement of the *reg6 *regenerating vessel phenotype by a histamine synthesis blocker

**Figure 6:**
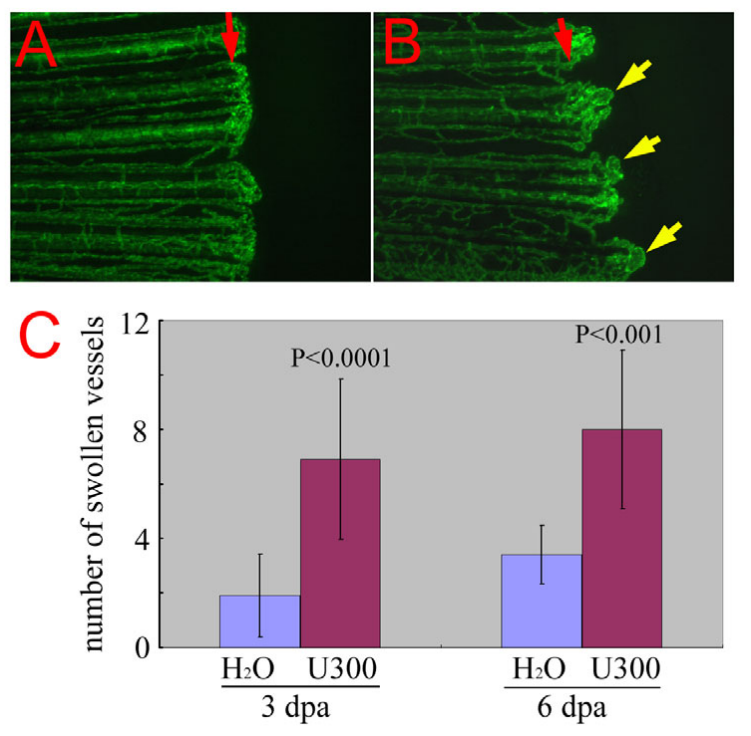
**Chemical enhancer of *reg6 *mutation**. (A) *reg6 *is a temperature-sensitive mutation and regenerates almost normally at 20°C. Shown is the regenerating vessels 6 days post amputation at 20°C. Note that they regenerated much more slowly at the low temperature. (B) When treated with 300 μM of the histidine decarboxylase inhibitor, urocanic acid, *reg6 *siblings developed a significant number of swollen vessels (yellow arrows). Red arrows, amputation plane. (C) The enhancement by urocanic acid of *reg6 *vessel defects was evaluated at two regenerative stages, during anastomosis (3 dpa at 20°C, *n *= 10) and plexus formation (6 dpa at 20°C, *n *= 10) that are both affected by the *reg6 *mutation [16]. U300, 300 μM of urocanic acid.

To further verify the role of histamine in promoting blood vessel branching, we next tested whether blocking histamine synthesis could enhance *reg6 *vessel branching defects. As stated before, the *reg6 *mutation is temperature sensitive. At a low temperature (20°C), the number of swollen vessels in *reg6 *regenerates was nearly zero (Figure 6). We treated *reg6 *regenerating vessels at this temperature with urocanic acid, one of the inhibitors of histidine decarboxylase which is the key enzyme for histamine synthesis. The results showed that the number of swollen vessels in 3-dpa *reg6 *regenerating fins at 20°C was about 1.9 ± 1.5/fin (*n *= 10), and this increased to 6.9 ± 2.9/fin (*n *= 9) with 300 μM urocanic acid (Figure 6A and 6B for morphological comparisons and C for quantitative data). A similar effect was also observed at 6 dpa during plexus formation of regeneration at 20°C (Figure 6C). Note that fin regeneration was much slower at 20°C than at 33°C, and regeneration at 3 and 6 dpa at 20°C corresponded to the vessel anastomosis and plexus formation stages of vessel regeneration, respectively, both of which are affected by the *reg6 *mutation [16]. Urocanic acid at 300 μM caused no *reg6*-like phenotype or toxicity in wild-type adult fish (data not shown).

### Histamine synthesis blocker causes blood vessel branching defect in wild-type zebrafish embryos

**Figure 7:**
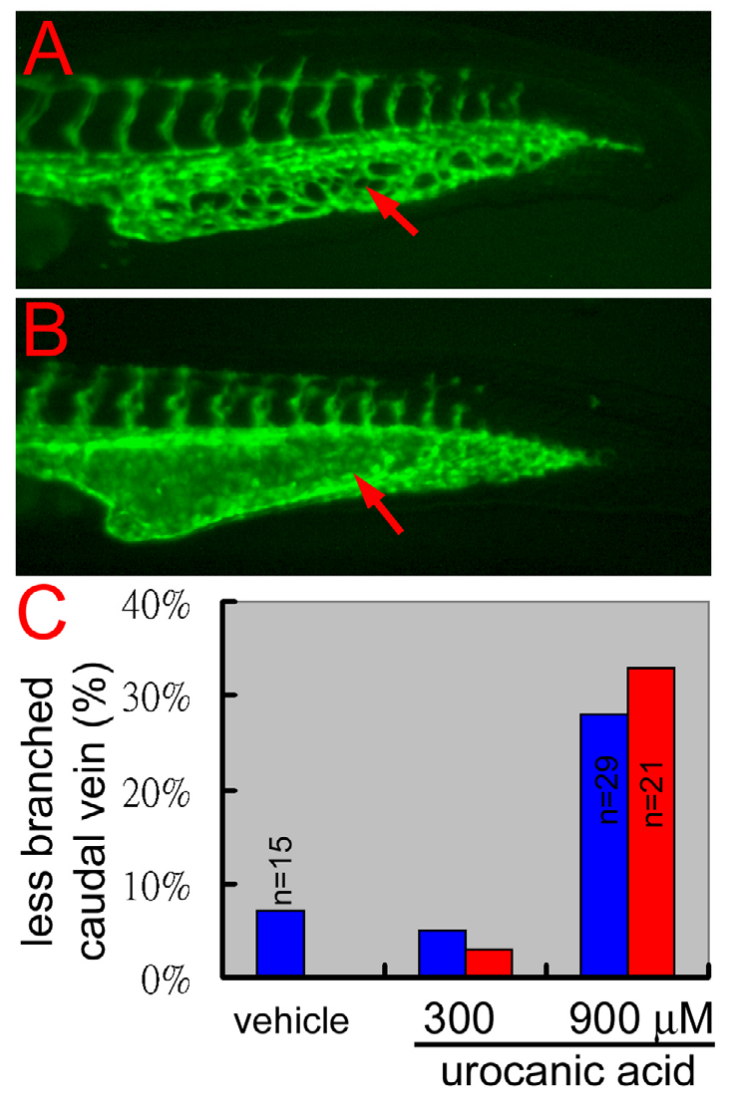
**Blocking of histamine synthesis causes blood vessel branching defects in wild-type zebrafish embryos**. (A) In a 32 hpf wild-type embryo, the developing caudal vein formed a transient vascular plexus. (B) When treated with 900 μM of the histamine synthesis blocker, urocanic acid, the caudal vein of a wild-type sibling formed much fewer vascular branches and even developed a *reg6*-like swollen lumen. Red arrows, caudal veins. (C) Quantitative results of the suppression of urocanic acid on blood vessel branching in wild-type zebrafish embryos. Two clutches of wild-type embryos (blue and red bars) were treated with the vehicle (0.0015 hydrochloric acid), or 300 or 900 μM of urocanic acid in egg water, from 26–36 hpf at 28.5°C. The percentage of embryos with less branched caudal vein was scored at the end of treatment (Y-axis, n = 40 unless otherwise specified).

To address whether histamine also influences blood vessel branching in wild-type fish, we treated the wild-type *TG(fli1:EGFP)*^*y*1 ^developing embryos with urocanic acid. In these experiments, we focused on the effect of urocanic acid on the caudal veins which normally form highly branched vascular plexuses during 30–48 hpf of the development (Figure 7A). Among the several doses and treating conditions that we tried, we found that 300 μM of urocanic acid, treated from 6–32 hpf, had no effect on the caudal vein development or the survival rate whereas 900 μM of urocanic acid could cause slightly increase of less branched caudal veins and more than 50% of lethality (data not shown). However, shorter treatment from 26–32 hpf with 900 μM urocanic acid caused lower lethality rate but significantly increase of poorly branched caudal veins (Figure 7B, C). These results demonstrate that blocking histamine synthesis indeed hampers blood vessel branching in wild-type zebrafish embryos.

### Association between egr-1 expression and the *reg6 *vascular phenotype

**Figure 8:**
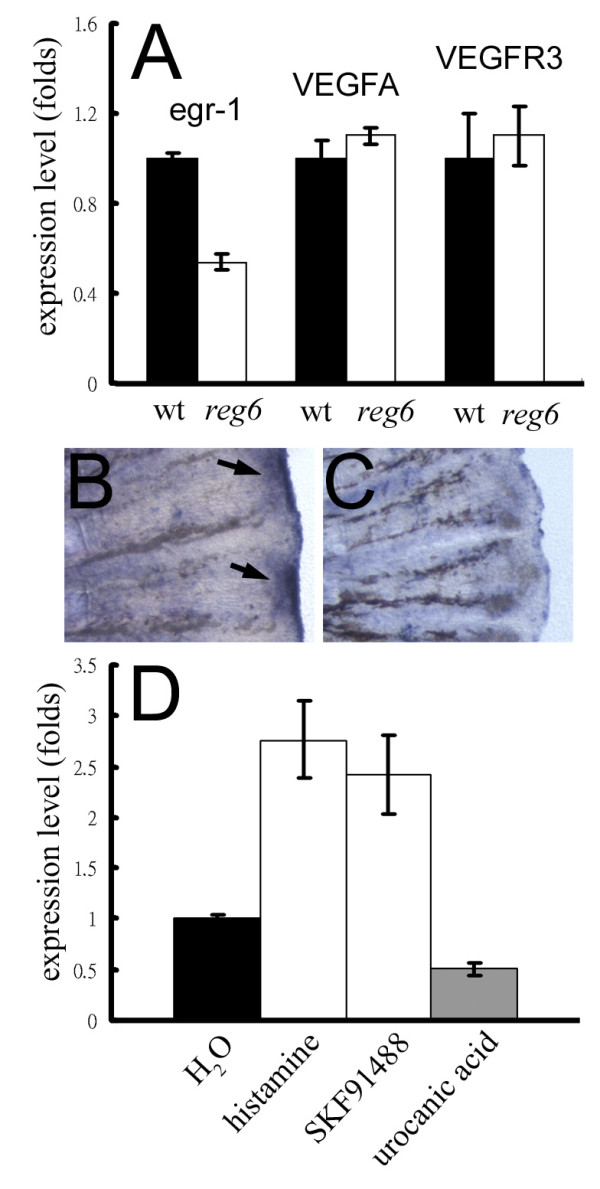
**Association of egr-1 expression and the *reg6 *vessel branching defect**. (A) Expression levels of the egr-1 gene in wild-type (wt; black bars) and *reg6 *(white bars) 3-dpa regenerates. (B, C) Whole-mount in situ hybridization for egr-1 on wild-type (B) and *reg6 *(C) 3-dpa regenerates showed decreased expression of egr-1 in *reg6 *fish. Arrows, egr-1 RNA signal. (D) The egr-1 expression in *reg6 *mutants was upregulated more than 2.5-fold by 1 mM histamine and 10 μM SKF91488 but was downregulated by 300 μM urocanic acid. The expression level was determined by QPCR and normalized by the expression level of the β-actin gene.

To test whether VEGF is involved in the mechanism for suppressing *reg6 *blood vessel branching defects by histamine, we performed QPCRs to examine the expression levels of known VEGFs and their receptors and some known angiogenic factors in wild-type and *reg6 *regenerating fins. We found no correlation between VEGF signaling and the *reg6 *phenotype, as the expression levels of zebrafish VEGF-A, C, and D, and VEGFR1, 2, and 3 were approximately the same in both wild-type and *reg6 *fish (Figure 8A and Additional file 3). Interestingly, we found that the level of egr-1, which is known to be involved in angiogenesis [20] was downregulated in *reg6 *fish (Figure 8A). Whole-mount in situ hybridization also showed a significant decrease in egr-1 expression in *reg6 *3-dpa regenerating fins (Figure 8C). More importantly, the expression of egr-1 in *reg6 *mutants was found to be elevated by approximately 2.5-fold after treatment with 1 mM histamine or 10 μM SKF91488 and downregulated by urocanic acid, compared with water-treated siblings (Figure 8D). In contrast, expression levels of VEGFs and their receptors did not change or changed by less than 2-fold after treatment with histamine, SKF91488, or urocanic acid (Additional file 4). These results suggest that histamine might promote blood vessel branching by regulating egr-1 expression directly or indirectly. Note that this correlation was only found in adult fish, not in embryos (data not shown), suggesting that histamine might influence regenerative and embryonic angiogenesis through different mechanisms.

## Discussion

### Zebrafish angiogenesis mutation for drug discovery and chemical genetics

We utilized a zebrafish mutation to demonstrate the power of zebrafish for drug discovery and examining chemical genetics. In particular, when using the embryonic phenotype for initial screening, which only required 0.5 μg of the test compound (with a MW of 250), the adult phenotypes, if they existed, allowed us to verify the drug effect with only 5 μg of drug for each test (see also "Methods"). In conjunction with *TG(fli1:EGFP)*^*y*1 ^transgenic fish, the screening could be specifically targeted for angiogenesis-related drugs (see also [21]). In addition, with zebrafish, we were able to perform toxicity tests at the same time at the whole organism level. All these factors make zebrafish a great tool for drug testing and discovery. The *reg6 *mutation is unique in that it is homozygously viable and displays similar blood vessel branching defects during embryogenesis and fin regeneration. More importantly, the *reg6 *mutation has the advantage of temperature sensitivity which allowed us to screen for suppressors and enhancers. Since *reg6 *mutants display specific defects in blood vessel branching, it has become a good tool for studying the genetic and pharmacological mechanisms of vessel branching which are largely unknown.

### The role of histamine in blood vessel branching in *reg6 *and wild-type zebrafish

Our results reveal a role of histamine in promoting vessel branching during angiogenesis using zebrafish *reg6 *mutants. The *reg6 *mutants were found to have specific defects in blood vessel branching during plexus formation [16]. When *reg6 *mutants were treated with the HMT inhibitor, SKF91488, which presumably causes accumulation of histamine, or with a high level of histamine, the blood vessel branching defect of *reg6 *was significantly rescued. In contrast, blocking histamine synthesis enhanced the *reg6 *phenotype. These results were further supported by the finding that HMT expression is elevated in *reg6 *mutants. All these suggest a simple model for the *reg6 *blood vessel branching defect which results from a high level of HMT and concomitant histamine deficit.

The role of histamine in regulating blood vessel branching is also observed in wild-type zebrafish. In Figure 7, we showed that blocking histamine synthesis with urocanic acid causes blood vessel branching defect and/or a *reg6*-like phenotype in the caudal veins of wild-type zebrafish embryos. In this experiment, however, we learned that it really required high concentration and careful selection of time window of urocanic acid treatment in order to see its effect on blood vessel branching. Treatment of high concentration of urocanic acid for long period of time, e.g. from 6–32 hpf caused high lethality rate in developing embryos suggesting that histamine is important for other developmental processes. Furthermore, we noted that urocanic acid did not seem to suppress the branching of intersegmental vessels, suggesting that the influence of histamine on vascular branching morphogenesis is selective or more sensitive for the plexus-forming blood vessels.

### Possible mechanisms of regulating blood vessel branching by histamine

As vascular branching involves complex intracellular and extracellular changes, including breakdown of the extracellular matrix and intercellular junctions, reorganization of the cytoskeleton, and induction of cell proliferation, it is difficult to predict the mechanism through which histamine rescues vessel branching defects in *reg6 *fish. Even though our phenotypic characterizations provide clues to the specific endothelial cellular defects underlying *reg6 *vascular defects, including defects in cell extension, cell junction formation, and likely permeability as well, it is still not possible at this moment to define a cellular mechanism for the rescue effect of histamine in *reg6 *fish due to the fact that the genetic identity of *reg6 *has yet to be resolved. We have mapped *reg6 *to the telomere region of LG 25 (CC Huang, unpublished results) where sequence information is largely lacking, and thus, it is difficult to obtain an accurate physical map. We are currently verifying a physical map of that region assembled by the Zebrafish Genome Sequencing Project at the Sanger Center with our *reg6 *mapping panels. We will need to build a more-accurate physical map of this region for future mapping and cloning of the *reg6 *gene.

Nevertheless, we have obtained more clues to *reg6*'s function from previous and the present studies. Our previous study showed that the *reg6 *mutation does not cause over-proliferation of regenerating endothelial cells and that *reg6 *function is specifically required during plexus formation when blood vessel endothelial cells are actively branching [16]. In this study, we further showed that the *reg6 *mutation does not affect the migration of regenerating endothelial cells. Instead, endothelial cells of *reg6 *mutants seem to be defective in extending cell processes and forming cell junctions among themselves. Our results also suggest that *reg6*'s function might be involved in regulating histamine metabolism and egr-1 expression. Consistent with previous results, we observed no effect of histamine on endothelial cell proliferation or migration in wild-type fish, even though it has been shown that histamine is able to cause endothelial cell proliferation in vitro [11,14]. Our studies using the zebrafish *reg6 *mutation provide evidence that histamine likely modulates blood vessel branching at a yet unidentified cellular level other than cell proliferation or migration.

### Correlation of egr-1 and the vessel branching defects of *reg6*

It is very intriguing that we found a close relationship between the egr-1 transcription factor and *reg6 *phenotypes. The level of egr-1 was low in *reg6 *mutant fish during regenerative angiogenesis, was elevated by histamine and an HMT inhibitor, and was further reduced upon treatment with a histamine synthesis blocker (Figure 8D). Although the role of egr-1 and its relationship with histamine in vascular branching awaits further studies, we found an interesting feature of egr-1 in our studies that seem to echo previous finding. Fahmy et al [20] has shown that egr-1 is required for angiogenesis and tumor growth through an FGF but not a VEGF signaling pathway. Our QPCR analyses showed that the transcription levels of VEGFs and VEGFRs remained the same in wild-type and *reg6 *fish with or without histamine manipulations suggesting that the mechanism(s) of blood vessel branching mediated by histamine that involves egr-1 is independent of the VEGF signaling. It would be exciting to know whether histamine modulates blood vessel branching directly through egr-1 and/or a FGF pathway.

### Direct and indirect actions of histamine on blood vessel endothelial cells

Histamine has been shown to increase vessel permeability by regulating the expression of cell adhesion molecules such as VCAM [15] and the composition of the extracellular matrix [22]. Presumably, these actions together can promote vessel branching. Our result that histamine can prevent the formation of swollen vessel lumina suggests its influence on actively branching vascular cells. We also showed that histamine can reverse the swelling of vessels and return them to normal, suggesting a role of histamine during vascular plexus remodeling. One possible explanation for these results is that histamine affects a cellular mechanism that is common to both vascular activities, for example cell adhesion or extracellular matrix remodeling. In fact, the histamine receptor inhibitor, cimitidine, has been shown to downregulate the endothelial expression of E-selectin adhesion molecules and prevent tumor metastasis [23], which raises the possibility that histamine might directly modulate vessel branching on endothelial cells by regulating E-selectin expression. However, the fact that histamine does not change endothelial cell proliferation or cause over-branching or any other morphological changes in wild-type fish suggests that endothelial cells are not the direct target of histamine. Thus, it is possible that histamine might both directly and indirectly influence vascular branching.

## Conclusion

We demonstrate the power of using a zebrafish mutation in pharmacogenetic studies. Our studies using the zebrafish *reg6 *mutation revealed the role of histamine in regulating blood vessel branching and the potential linkage of angiogenic factor egr-1 to this process.

## Methods

### Fish husbandry

Fish maintenance and breeding were performed according to standard procedures [24].

### Chemical screening with *reg6 *embryos

*reg6 *homozygous embryos were obtained by in vitro fertilization for chemical screening of clones due to the concerns that *reg6 *embryos from different clutches might show variable degrees of phenotypic penetrance. Embryos from different pairs of parents were kept separate, and 20 embryos from each individual clutch were set aside to monitor the phenotypic penetrance. The protocol for chemical treatment of zebrafish embryos was adapted from Peterson et al. [25] with minor modifications. These embryos were allowed to develop at 28.5°C until 6 h post-fertilization (hpf) and then were arrayed into 96-well plates, at 5 embryos/well with 200 μl of egg water (distilled water containing 60 μg/ml sea salt) without antibiotics. The LOPAC chemical library (Sigma, St. Louis, MO, USA) was prepared at 2 mM in DMSO, and 1 μl of each was added to each well to make 10 μM for our initial screening. These plates were kept in a 33°C incubator and examined at around 32 hpf with a fluorescent stereomicroscope. Chemicals used in this study were purchased from Sigma unless otherwise signified.

### Chemical treatment of adult fish

Adult fish were briefly anesthetized with 0.01% tricaine, and the caudal fins were amputated vertically at 50% of the proximal-distal axis. Five amputated fish were put into a 250-ml beaker containing 100 ml of aquatic water with or without chemicals. These fish were then kept in a 33°C incubator for 3 days without feeding. With this protocol, the wild-type fish could regenerate normally, while *reg6 *fish developed swollen vessel lumina. To quantify the *reg6 *phenotype, the number of vessels that developed obviously swollen lumina was determined. Since each fin ray except for the two lateral fin rays contains three vessels [16], 48 vessels of the 16 fin rays were examined and scored for a typical caudal fin with 18 fin rays.

### Transmission electron microscopy

Embryos were fixed in 2% glutaraldehyde, 2% paraformaldehyde, and 0.1 M sodium phosphate (pH 7.4) overnight at 4°C. Fixed embryos were washed with 0.1 M sodium phosphate buffer 3 times for 5 min each, and post-fixed with 1% OsO_4 _for 1 h at room temperature. The fixed embryos were dehydrated by the following ethanol series: 30% for 10 min, 50% for 10 min, 70% for 20 min, 80% for 20 min, 90% for 20 min, 95% for 20 min, and 100% for 60 min, and then twice in acetone for 30 min each. Infiltration was carried out first with 1:1 resin: acetone for 1 h, 2:1 resin: acetone for 1 h, pure resin for 1 h, and finally pure resin overnight. Embryos were placed in a 70°C oven overnight for embedding. The plastic blocks were then trimmed and sectioned to obtain 1 μm or ultrathin section of 70~90 nm which were collected on 200-mesh copper grids and stained with uranyl acetate for 50 min and lead citrate for 10 min. Sections were analyzed under a Hitachi H-7000 transmission electron microscope equipped with AMT XR40 CCD camera (Advanced Microscopy Techniques, Danvers, MA USA).

### Whole mount *in situ *hybridization

Regenerative fins were harvested and fixed in 4% paraformaldehyde overnight at 4°C. The whole-mount *in situ *hybridization was done with the InsituPro VS robot by Intavis Bioanalytical Instruments AG (Koln, Germany) following standard procedures of the manufacturer.

### Quantitative polymerase chain reaction (QPCR)

Wild-type or *reg6 *homozygous embryos were treated with chemicals for 26 h (from 6 to 32 hpf) or 8 h (from 24 to 32 hpf) at 33°C and then manually dechorionated at the end of treatment. Fifty embryos from each treatment were subjected to RNA extraction with Trizol following instructions of the manufacturer. Around 5 μg of total RNA was then used to generate cDNAs with Superscript II (Invitrogen). Quantitative PCR was performed with a Power Cybergreen (ABI) labeling kit in an ABI7000 thermocycler. The data were analyzed with the software provided by ABI. When using adult fish, five 3-dpa regenerative fins from treated or untreated wild-type or *reg6 *fish were collected.

Primers used for quantitative PCR were egr-1F, AGTTTGATCACCTTGCTGGAGATAC; egr-1R, AGGGTGAAACGGCCTGTGT; HMT-F, AATGAAGTGGTGGAACCA AGTAAT G; HMT-R, AAGATCGGGAGATGTTGACACTCT; VEGF-A-F, GCTGTAAAGGCTGCCCACATAC; VEGF-A-R, ACCAGCAGCTCTCGGGTCTT; VEGF-C-F, GCGGACCACACCATTACC; VEGF-C-R, TGCGGTTGAGAGGTTGAC; VEGF-D-F, AAAGAGGGAGTTACCTGCCGTAAT; VEGF-D-R, AGCACAGGCTCTGGTCCAGATA; VEGFR1-F, GTCACTAACCCAGACGCCAAAG; VEGFR1-R, ATGAATCCCTGCCTGCTGTT; VEGFR2-F, TCTTCACTCTTCACGTGCTTTTTAG; VEGFR2-R, GAAGGTGTGTATCTCCATCAGGAA; VEGFR3-F, ACTGTCGGCCGTGTGGTTA; and VEGFR3-R, CGAATCCTTCAGGGATAGTGGTT.

## Authors' contributions

CCH designed and carried out most of the experiments. CWH performed the QPCR and whole-mount in situ hybridization experiments. YSEC provided the chemical library and helped design the chemical screening experiment. CCH and JY examined the data and wrote the paper. All authors read and approved the final manuscript.

## Supplementary Material

Additional file 1 TEM image of the regenerating blood vessels of wild-type fish. A representative TEM image of a wild-type 3-dpa regenerate shows vascular plexus evident by the narrow canal (black arrow) and connection (white arrow) between blood vessels that are of different sizes. B, blood cell; E, endothelial cell; BV, blood vessel. Scale bar, 2 μm. Click here for file

Additional file 2 TEM images of the regenerating blood vessels of *reg6 *fish. Composite TEM images of a *reg6 *3-dpa regenerate show enlarged blood vessel (lower left cornor) which is filled with blood cells. Blood cells are often leaked out of the blood vessels (arrows). The endothelial cells in *reg6 *are less stretch-out and sometimes stacked (arrowhead). However, no clear cell junction is found among these cells. B, blood cell; E, endothelial cell. Scale bar, 2 μm. Click here for file

Additional file 3 Expression levels of VEGF related genes in wild-type and *reg6 *regenerative fins. The expression levels of VEGFC, VEGFD, VEGFR1, and VEGFR2 is about the same in wild-type (black bars) and *reg6 *(white bars) 3-dpa regenerates. Note that the level of VEGFC seems higher but the magnitude is less than two folds. The expression level is determined by QPCR and normalized by the expression level of β-actin gene. Click here for file

Additional file 4 Expression levels of VEGF related genes in *reg6 *regenerative fins upon different treatments. The treatment of (1) H_2_O, (2) 1 mM histamine, (3) 10 μM SKF91488, or (4) 300 μM urocanic acid does not alter significantly the expression levels of VEGF-A, VEGF-C, VEGF-D, VEGFR1, VEGFR2, and VEGFR3 in *reg6 *3-dpa regenerates. Click here for file
