# Supplementary figures and images for: Histamine metabolism influences blood vessel branching in zebrafish reg6 mutants
Source: BMC Dev Biol. 2008 Mar 25;8:31. doi: 10.1186/1471-213X-8-31 (PMC2291033; doi:10.1186/1471-213X-8-31)

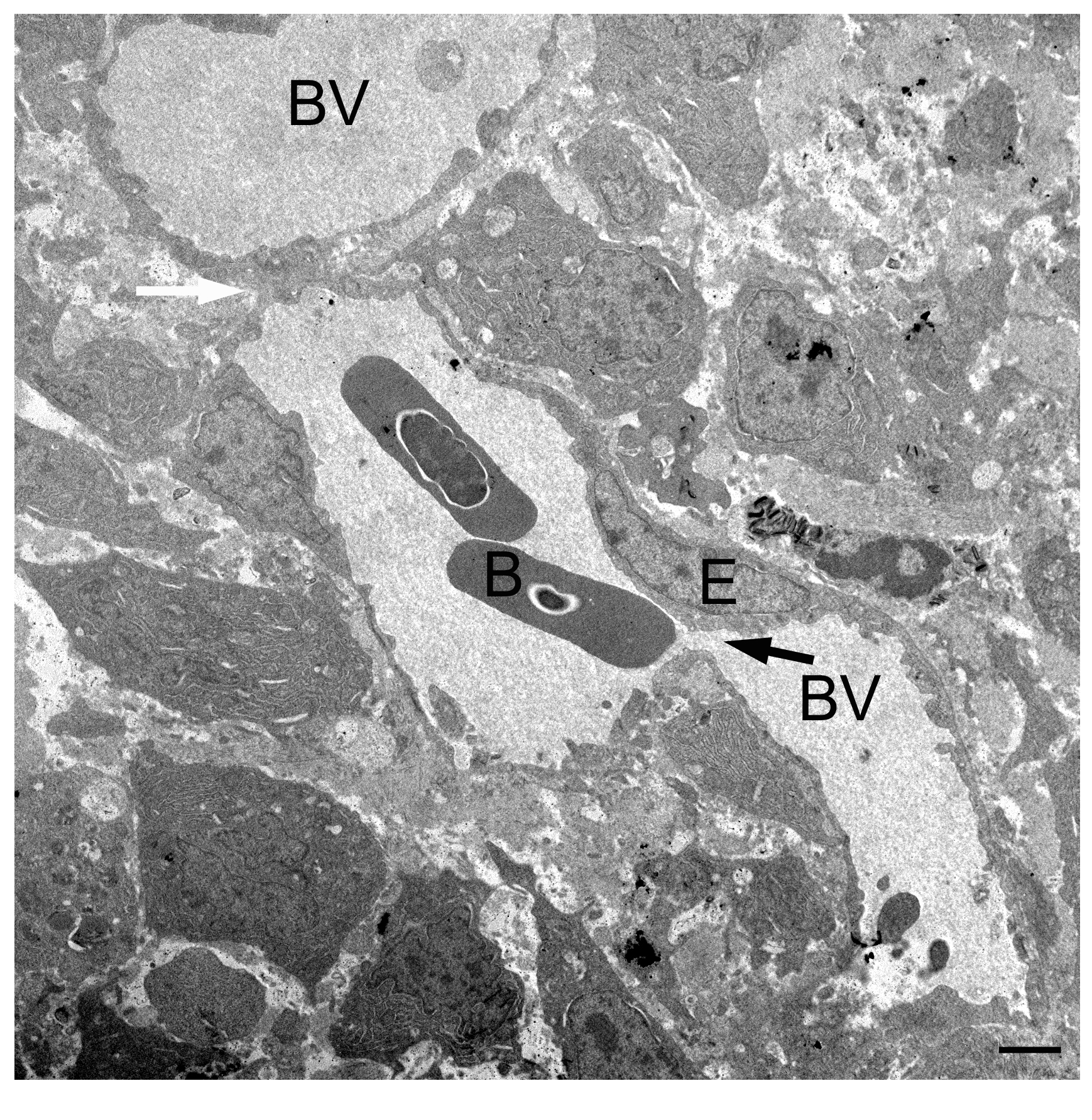

Supplement: Additional file 1 — TEM image of the regenerating blood vessels of wild-type fish. A representative TEM image of a wild-type 3-dpa regenerate shows vascular plexus evident by the narrow canal (black arrow) and connection (white arrow) between blood vessels that are of different sizes. B, blood cell; E, endothelial cell; BV, blood vessel. Scale bar, 2 μm. [file 1471-213X-8-31-S1.jpeg]

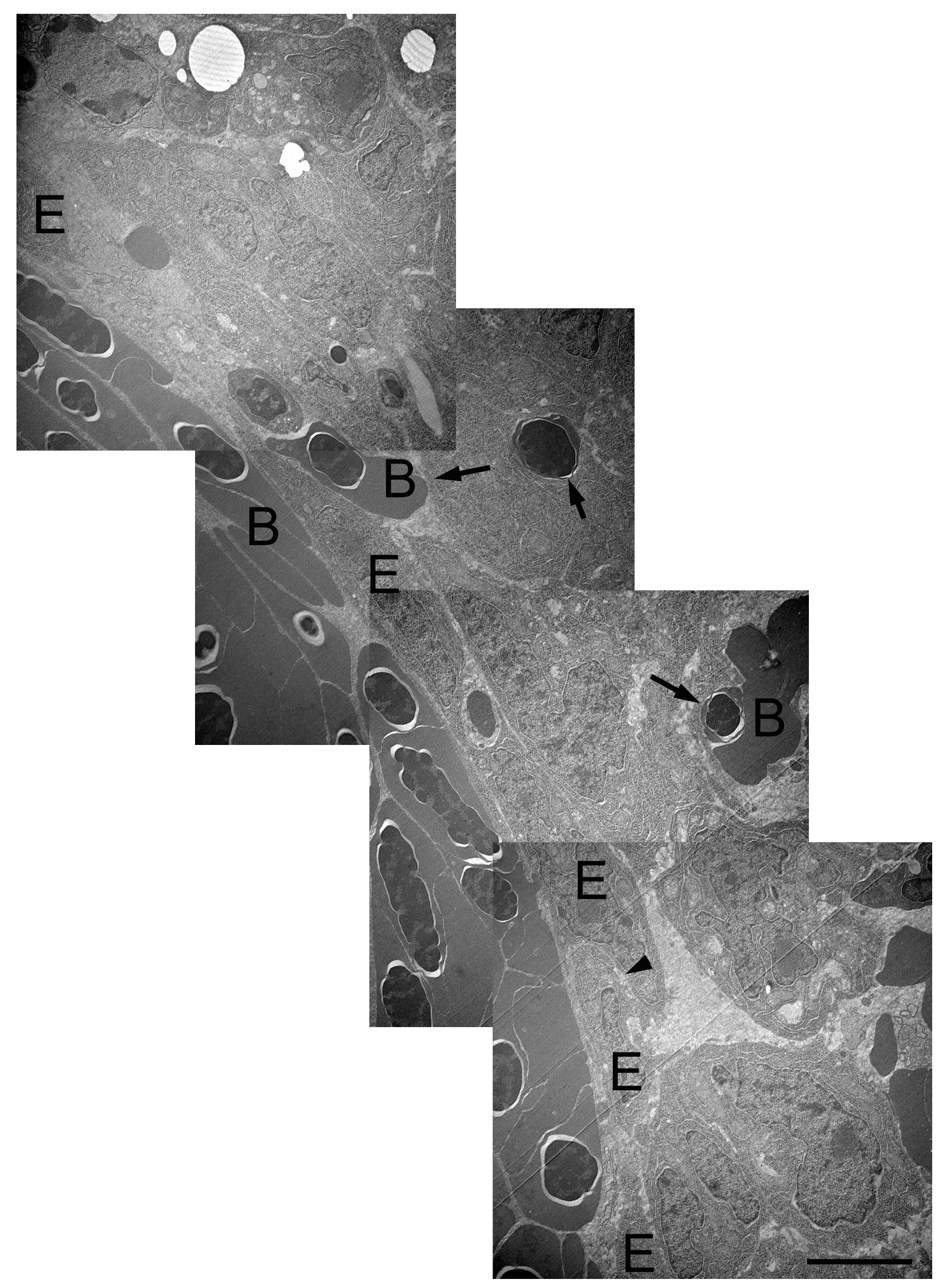

Supplement: Additional file 2 — TEM images of the regenerating blood vessels of reg6 fish. Composite TEM images of a reg6 3-dpa regenerate show enlarged blood vessel (lower left cornor) which is filled with blood cells. Blood cells are often leaked out of the blood vessels (arrows). The endothelial cells in reg6 are less stretch-out and sometimes stacked (arrowhead). However, no clear cell junction is found among these cells. B, blood cell; E, endothelial cell. Scale bar, 2 μm. [file 1471-213X-8-31-S2.jpeg]

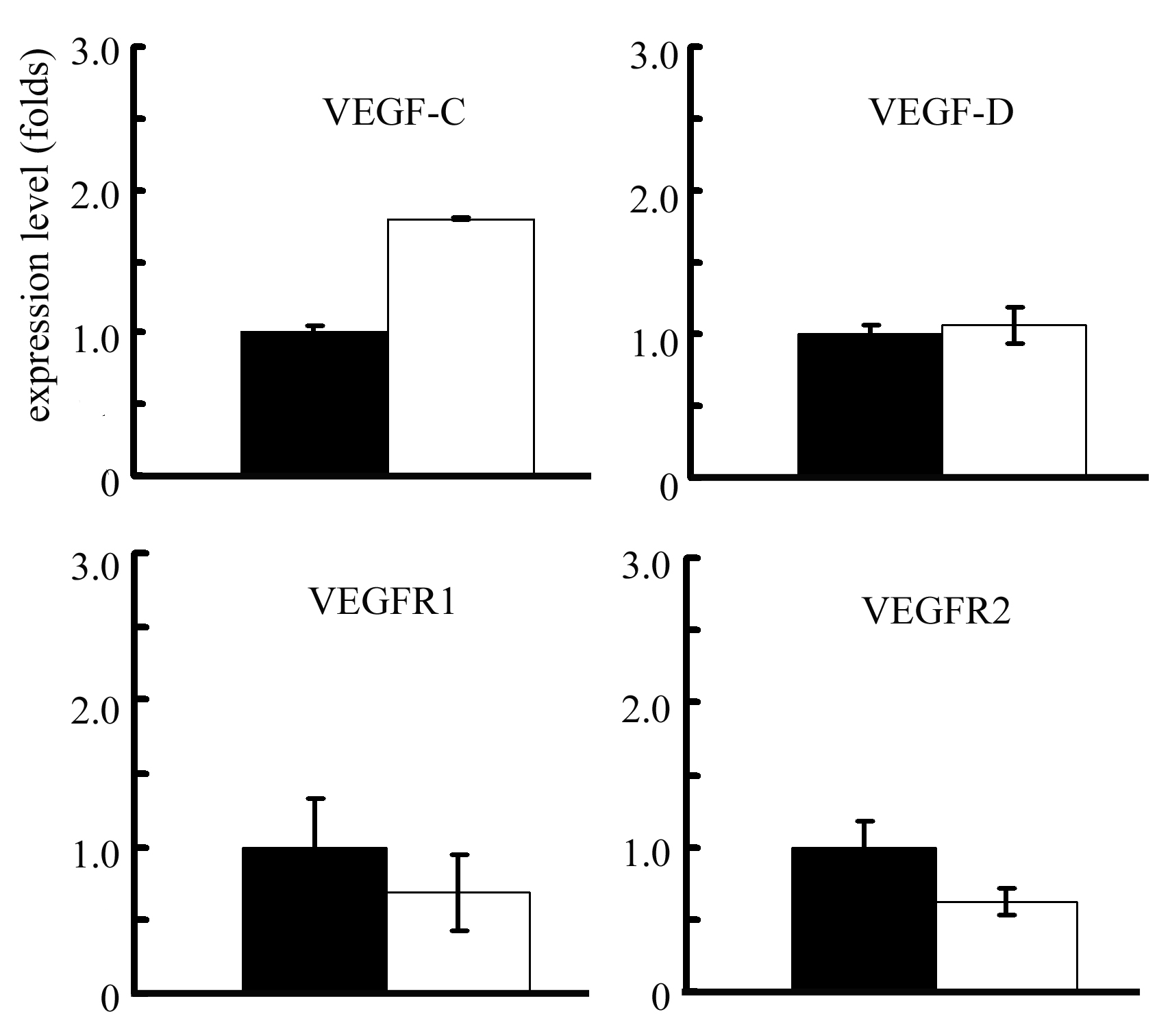

Supplement: Additional file 3 — Expression levels of VEGF related genes in wild-type and reg6 regenerative fins. The expression levels of VEGFC, VEGFD, VEGFR1, and VEGFR2 is about the same in wild-type (black bars) and reg6 (white bars) 3-dpa regenerates. Note that the level of VEGFC seems higher but the magnitude is less than two folds. The expression level is determined by QPCR and normalized by the expression level of β-actin gene. [file 1471-213X-8-31-S3.jpeg]

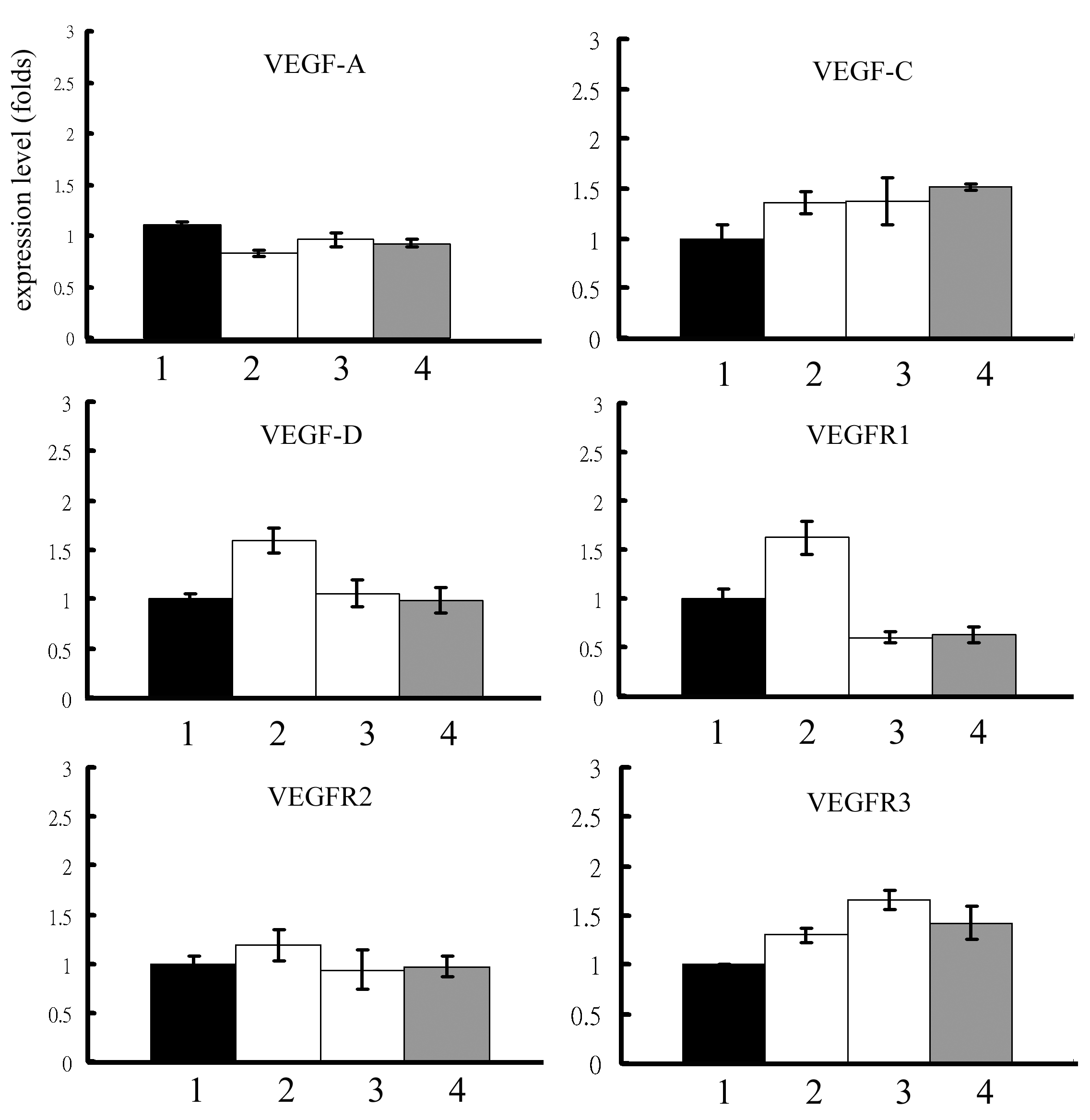

Supplement: Additional file 4 — Expression levels of VEGF related genes in reg6 regenerative fins upon different treatments. The treatment of (1) H2O, (2) 1 mM histamine, (3) 10 μM SKF91488, or (4) 300 μM urocanic acid does not alter significantly the expression levels of VEGF-A, VEGF-C, VEGF-D, VEGFR1, VEGFR2, and VEGFR3 in reg6 3-dpa regenerates. [file 1471-213X-8-31-S4.jpeg]
